# Effective Chemical Lift-Off for Air-Tunnel GaN on a Trapezoid-Patterned Sapphire Substrate

**DOI:** 10.3390/mi14040753

**Published:** 2023-03-29

**Authors:** Min-joo Ahn, Kyu-yeon Shim, Woo-seop Jeong, Seongho Kang, Hwayoung Kim, Seunghee Cho, Dongjin Byun

**Affiliations:** 1 Department of Materials Science and Engineering, Korea University, 145 Anam-ro, Seongbuk-gu, Seoul 02841, Republic of Korea; 2 Semiconductor R&D Group, Wonik IPS, 75 Jinwisandan-ro, Jinwi-myeon, Pyeongtaek-si 17790, Republic of Korea

**Keywords:** gallium nitride, chemical lift-off, trapezoid-patterned sapphire substrate

## Abstract

We fabricated an air-tunnel structure between a gallium nitride (GaN) layer and trapezoid-patterned sapphire substrate (TPSS) through the in situ carbonization of a photoresist layer to enable rapid chemical lift-off (CLO). A trapezoid-shaped PSS was used, which is advantageous for epitaxial growth on the upper c-plane when forming an air tunnel between the substrate and GaN layer. The upper c-plane of the TPSS was exposed during carbonization. This was followed by selective GaN epitaxial lateral overgrowth using a homemade metal organic chemical vapor deposition system. The air tunnel maintained its structure under the GaN layer, whereas the photoresist layer between the GaN layer and TPSS disappeared. The crystalline structures of GaN (0002) and (0004) were investigated using X-ray diffraction. The photoluminescence spectra of the GaN templates with and without the air tunnel showed an intense peak at 364 nm. The Raman spectroscopy results for the GaN templates with and without the air tunnel were redshifted relative to the results for free-standing GaN. The CLO process using potassium hydroxide solution neatly separated the GaN template with the air tunnel from the TPSS.

## 1. Introduction

GaN is a promising III–V semiconductor used in light-emitting diodes (LEDs), laser diodes, and high-power transistors, owing to its wide and direct bandgap. Among these applications, GaN-based compound semiconductors are generally used in LEDs owing to their advantage of covering the wavelength range that emits ultraviolet and infrared rays as well as the visible light range [1,2]. The internal quantum efficiency (IQE) and light extraction efficiency (LEE) of GaN-based LEDs can be enhanced by utilizing a patterned sapphire substrate (PSS) technique [3,4,5]. It is well-established that, as a mask-free approach, the PSS technique redirects photons reaching the patterned substrate to the surface of the device and reduces the threading dislocation density [6,7,8]. Decreasing the threading dislocation density is beneficial for improving the IQE because the threading dislocations act as non-radiative recombination centers [9,10]. Selective area growth, which is suitable for a PSS with various planes, reduces the threading dislocation density at the bottom layer of the PSS by creating a template with epitaxial lateral overgrowth (ELOG) [11,12,13]. The ELOG process can significantly reduce defects such as threading dislocations caused by the large lattice mismatch and difference in the thermal expansion coefficient of the sapphire substrate and GaN film [14,15].

The epitaxially grown template and sapphire substrate were separated to improve heat dissipation and LEE [16,17,18]. Two common lift-off methods are used to separate the epitaxial layer and sapphire substrate of an LED chip, namely laser (optical) lift-off (LLO) and chemical lift-off (CLO). The LLO technique utilizes lasers absorbed at the interface between the GaN layer and sapphire, which induces the rapid thermal decomposition of GaN [19,20]. This technique has the advantages of being relatively faster than CLO and enabling precise separation due to selective light absorption between the epitaxial layer and substrate [21]. However, the LLO technique using intense irradiation has the disadvantages that thermal defects arise from the instantaneous generation of high temperature and that the GaN film deforms due to the pressure of decomposed N_2_ gas [22]. By contrast, the CLO technique selectively etches away the sacrificial layer and separates the substrate from the epilayer. CLO can potentially replace LLO, as it has a relatively lower cost in commercial applications [23]. In addition, using a batch system has the advantage of higher production yields because numerous wafers can be processed under a single CLO process. However, the CLO technique has the disadvantage of requiring a long time to etch the interface in all directions and separate epilayers through chemical reactions [23,24,25]. In addition, the materials used for the sacrificial layer must have a lattice match with the substrate and epilayer [24,26]. The fabrication of a GaN template with an air-tunnel structure for a rapid CLO process was investigated in our previous study. Jeong et al. [27] introduced a structural design for the CLO process using a sacrificial layer and relatively complex processes such as oxygen plasma treatment and ex situ sacrificial layer deposition.

Therefore, in this study, to overcome the aforementioned disadvantages of CLO, we propose a structure for achieving effective etchant penetration. We fabricated an air-tunnel structure between the GaN epilayer and TPSS through the carbonization of the photoresist layer. A wide air tunnel is linked to the entire GaN/TPSS interface to induce rapid diffusion and etching without a sacrificial layer, followed by separation of the substrate without damaging GaN. Fabrication of the air-tunnel structure and GaN growth are conducted in the same metal organic chemical vapor deposition (MOCVD) reactor. The photoresist is carbonized, and GaN is grown in a selective area to fabricate a GaN template with an air tunnel. The crystallinity, optical properties, and internal stress of the grown GaN with and without an air tunnel are analyzed.

## 2. Materials and Methods

**Figure 1:**
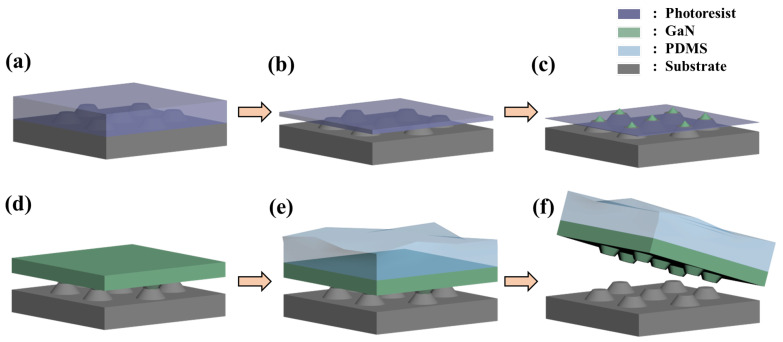
Schematic of the process for fabricating GaN epitaxial lateral overgrowth with air tunnel; (**a**) spin coating of photoresist, (**b**) carbonization of photoresist, (**c**) GaN selective area growth, (**d**) GaN epitaxial lateral overgrowth, (**e**) PDMS coating to enable handling, and (**f**) CLO with KOH solution.

A trapezoid-shaped PSS (0001) was prepared to fabricate an air tunnel between the substrate and GaN layer. For the experiments, the TPSS wafer was cut to a size of 1 cm × 1 cm. The height, top diameter, and bottom diameter of each trapezoid pattern were 1.15 μm, 1.55 μm, and 2.65 μm, respectively. The experiments consisted of the following steps, as shown in Figure 1a–f. Figure 1a illustrates the photoresist spin-coating process, which was performed twice at various spin speeds. An AZ-HKT-501 photoresist was used in this experiment. Subsequently, the spin-coated photoresist layer was baked on a hot plate at 150 °C to vaporize the solvent. A homemade MOCVD system was employed for the carbonization of the photoresist and growth of GaN. Figure 1b presents the in situ photoresist carbonization process, which was conducted in an MOCVD reactor for 5 min under hydrogen gas (H_2_, 99.999%) atmosphere (1100 °C and 45 Torr). The volume of the photoresist layer vertically decreased. Subsequently, selective area growth of the GaN template was continued with trimethylgallium (TMGa) as the Ga source, ammonia (NH_3_) gas as the N source, and H_2_ as the carrier gas, under vertical growth conditions (1020 °C and 300 Torr), as depicted in Figure 1c. The thickness of the photoresist simultaneously decreased during the vertical growth of GaN. Lateral overgrowth was performed under lateral growth conditions (1100 °C and 45 Torr) after the vertical GaN growth, as shown in Figure 1d. Further experiments were performed to examine the CLO of GaN. Polydimethylsiloxane (PDMS) was coated on the surface to handle the thin GaN films, as illustrated in Figure 1e. Figure 1f shows the CLO process for separating the substrate and GaN layers. The GaN template coated with PDMS was immersed in 1 M KOH solution at 80 °C.

The schematic of the experimental process in Figure 1 was confirmed from the scanning electron microscope (SEM) images. The crystal orientation of the GaN template grown with an air tunnel was analyzed using XRD. PL and Raman spectroscopy were used to analyze the optical properties and internal stress of the grown GaN.

## 3. Results and Discussion

In the case of a GaN layer grown on a bare sapphire substrate with a flat c-plane, for an etchant to pass through the entire interface of the GaN/sapphire template during the CLO process is ineffective. Therefore, in this experiment, we proposed a method of forming a wide space at the GaN/sapphire interface to allow the etchant to penetrate the interface, which induces a high etching rate during the CLO process. PSSs used for GaN growth have various shapes, such as conical, trapezoidal, and hemispherical [28,29]. Of these, a trapezoidal pattern that could form an air tunnel with sufficient height and selectively grow GaN on the c-plane was considered the most suitable substrate for our experiments [26,30]. We used a trapezoid-shaped PSS to form an air tunnel between the substrate and GaN layer during GaN film deposition. Unlike the common process of GaN epitaxial overgrowth using the PSS technique, which induces lateral overgrowth after vertical growth on the lower c-plane of the PSS [28,31], we induced GaN growth on the upper c-plane while inhibiting it on the lower c-plane of the TPSS. Consequently, an air tunnel was formed between the lower c-plane of the TPSS and GaN layer.

**Figure 2:**
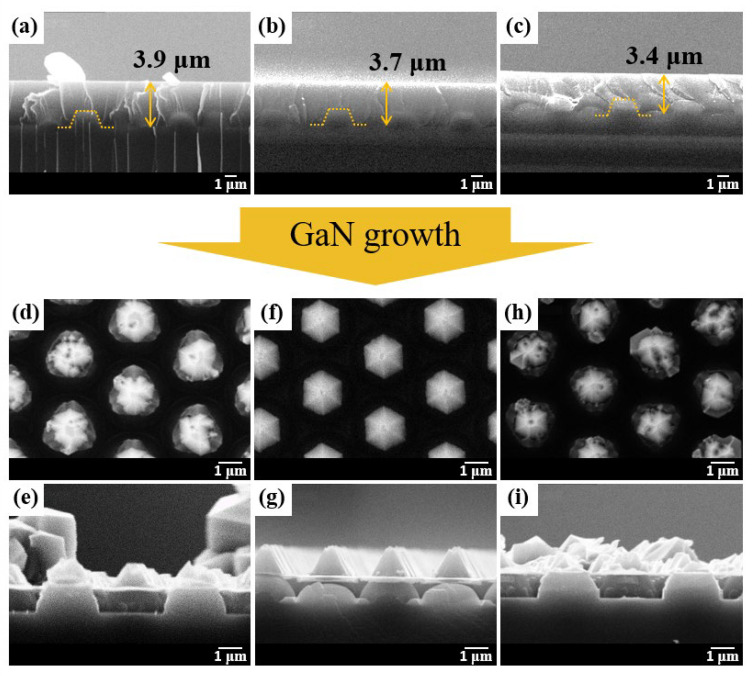
Cross-sectional SEM images of photoresist layers spin-coated at speeds of (**a**) 3200 rpm, (**b**) 3700 rpm, and (**c**) 4200 rpm. The dashed lines and arrows represent the TPSS lens and the thickness of photoresist layer, respectively. Top and cross-sectional SEM images of GaN grown on sample spin-coated at (**d**,**e**) 3200 rpm, (**f**,**g**) 3700 rpm, and (**h**,**i**) 4200 rpm.

Figure 2a–c show the cross-sectional SEM images of the photoresist layers spin-coated on the TPSS at different spin-coating speeds for optimizing the photoresist coating conditions to form an air tunnel. Figure 2a–c show the images of the photoresist layers spin-coated at speeds of 3200, 3700, and 4200 rpm, respectively, with corresponding thicknesses of 3.9, 3.7, and 3.4 μm. Figure 2d–i show the top and cross-sectional SEM images of GaN grown for 5 min after the carbonization of each photoresist layer at different spin-coating speeds. During the carbonization of the photoresist in the MOCVD reactor at high temperatures, the spin-coated photoresist undergoes thermal contraction upward and downward based on the horizontal plateau surface of the TPSS. Additionally, an air tunnel is formed between the photoresist layer and substrate via thermal contraction. The SEM image following the carbonization of the photoresist layer demonstrated a more contracted shape than the shape when GaN was grown immediately after carbonization; therefore, it was not included in the text to avoid confusion. Figure 2d,e show the top and cross-sectional SEM images, respectively, of GaN grown on the sample spin-coated at 3200 rpm. It was speculated that the photoresist remained on the upper c-plane of the TPSS immediately after carbonization because GaN did not grow in a hexagonal pyramidal shape. In contrast, the top and cross-sectional SEM images of GaN grown on the sample spin-coated at 4200 rpm demonstrate that the carbonized photoresist layer has a lower height than the upper c-plane of the TPSS (Figure 2i). Additionally, GaN growth occurred on surfaces other than the c-plane (Figure 2h). As shown in Figure 2f,g GaN grown on the sample spin-coated at 3700 rpm underwent selective area growth, with a hexagonal pyramid-shaped morphology. Only the upper c-plane of the TPSS was exposed, where the photoresist was not present. In contrast, the photoresist remained on the surface except for the upper c-plane of the TPSS. A wide air tunnel with a height of 900 nm was formed between the photoresist and substrate, and an approximately 300 nm thick photoresist layer was observed on the horizontal plateau surface of the TPSS. Despite the presence of the photoresist, GaN grew selectively on the upper c-plane of all patterns. Consequently, GaN can be grown in a selective area using the photoresist carbonization process. Considering the thermal contraction of the photoresist at high temperatures, the optimal height of the photoresist layer is crucial, as the height of the photoresist can suppress growth on the bottom c-plane of the TPSS while allowing the vertical growth of GaN on the top c-plane of the TPSS. Under the spin-coating conditions presented in Figure 2b, the sample spin-coated at 3700 rpm showed suitable vertical GaN growth, which is consistent with the aforementioned optimized height.

**Figure 3:**
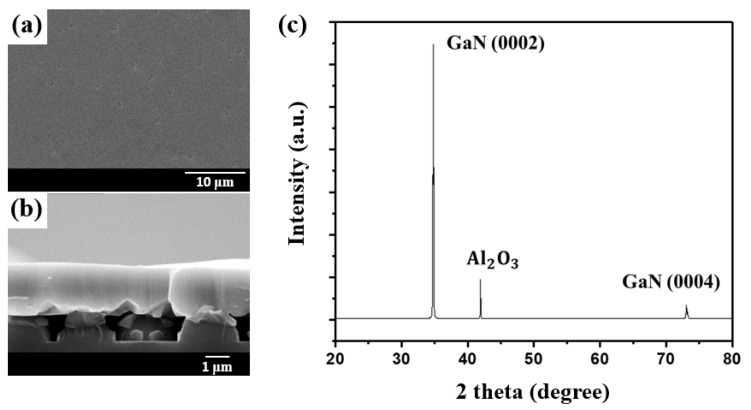
Top (**a**) and cross-sectional (**b**) SEM images of GaN template; (**c**) XRD pattern of GaN template with air tunnel.

Figure 3a,b show the top and cross-sectional SEM images, respectively, of GaN that was laterally grown for 30 min after the vertical growth process shown in Figure 2. Figure 3a, which is the SEM image of the upper part of the grown GaN film, shows that all regions were grown by merging into the film despite a rather short growth time. A GaN film with a thickness of 2 μm was successfully grown using the ELOG method, as shown in Figure 3b. In addition, Figure 3b indicates that GaN started to grow in the plateau region of the TPSS, and an air tunnel with a width of 1 μm or higher was formed under the GaN template. Some GaN particles were present on the PSS lens in the air tunnel, which are believed to have grown there as a result of the rapid etching of the photoresist in some parts. In addition, no trace of photoresist could be identified. The crystallinity of the GaN film with an air tunnel was examined using XRD, as shown in Figure 3c. The diffraction patterns show peaks corresponding to the (0002) and (0004) planes of GaN. The sharp (0002) peak in the XRD pattern of GaN was located at 34.54°, and GaN (0004) was observed at 73.02°. The XRD analysis detected no additional materials than GaN and the sapphire substrate.

**Figure 4:**
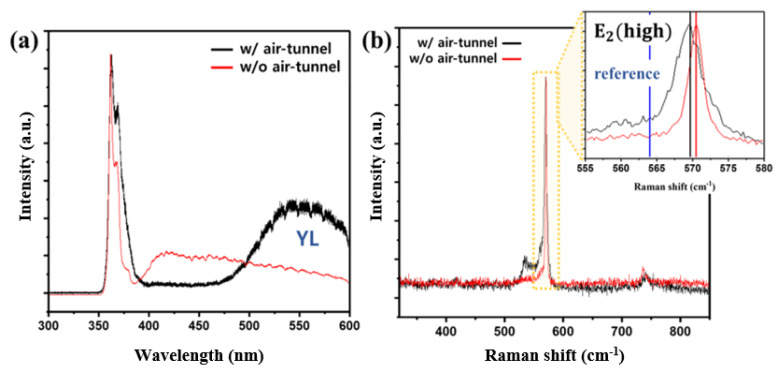
(**a**) PL spectra of GaN films with/without an air tunnel at 300 K. (**b**) Raman spectroscopy of GaN films with/without an air tunnel.

The optical characteristics of the GaN films with air tunnels were analyzed using PL spectroscopy. For comparison, a GaN epilayer reference sample with a thickness of 2 μm was grown using MOCVD on c-plane bare sapphire under the same growth conditions. Figure 4a illustrates the visible range of the PL spectra of the GaN films with and without an air tunnel. The spectra of both samples exhibited near-band edge luminescence with an intense peak at 364 nm, which was assigned to donor-bound exciton ($D^{0}X$) [32,33]. In addition, intense broadband yellow luminescence spanning 500–600 nm was observed in the case of the GaN template containing the air tunnel, representing deep-level sites such as internal GaN structural defects. This suggested that the GaN template with the air tunnel affected the GaN internal defects because of the carbon impurities generated during carbonization. A photoresist with a thickness of approximately 300 nm was speculated to be present at the onset of GaN growth, which may cause carbon contamination and carbonization; it can also affect vertical GaN growth by producing carbon impurities inside GaN. In addition, these carbon impurities may cause dislocations inside the GaN film with an air tunnel [34,35,36]. Raman spectroscopy was used to measure the internal stress of GaN with and without an air tunnel, as shown in Figure 4b. Raman spectra were generated in the backscattering mode at room temperature, and laser light was incident along the normal direction of the grown GaN samples. The excitation wavelength was 532 nm. Both sample geometries showed the same peaks, with the $A_{1}$ (LO) and $E_{2}$ (high) modes at 733.89 cm^−1^ and approximately 570 cm^−1^, respectively. By contrast, the geometry of the GaN template with an air tunnel showed different peaks than those of the GaN template without an air tunnel, with the $\;A_{1}$ (TO) and $E_{1}$ (TO) modes at 532.11 cm^−1^ and 567.56 cm^−1^, respectively. In the GaN film with an air tunnel, the $E_{2}$ (high) mode occurred at 569.7 cm^−1^, and the $E_{2}\;$(high) mode of the GaN film without an air tunnel was observed at 570.6 cm^−1^. The $E_{2}\;$(high) modes of both samples exhibited a redshift as compared with the $E_{2}\;$(high) mode position (564 cm^−1^) of free-standing GaN [37]. Both samples appeared to have compressive stress; however, the GaN template containing the air tunnel had less compressive stress than the GaN template without the air tunnel. Although it was expected that the internal stress would be low owing to the ELOG, it is suggested that the internal stress was caused by lattice mismatch owing to the carbon impurities generated during carbonization.

**Figure 5:**
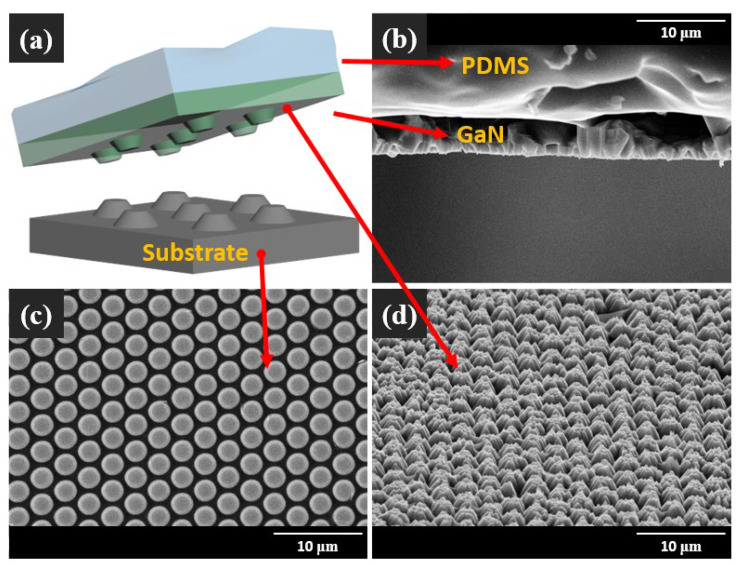
(**a**) Schematic of the CLO process between GaN and substrate with KOH solution, SEM measurements showing each zone after CLO, (**b**) cross-sectional SEM image of GaN film with PDMS, (**c**) top view of the SEM image of the substrate, and (**d**) bird’s eye view of the SEM image depicting the interface between GaN and the substrate.

The GaN template with the air tunnel was then separated from the substrate. Subsequently, PDMS was applied to the surface of the GaN film with an air tunnel, and CLO was performed by immersing the film in KOH solution [38,39]. Figure 5a shows a schematic of the CLO data for the GaN film and TPSS. The air tunnel allowed the KOH solution to separate the GaN from the substrate in approximately 5 h. Figure 5b presents a cross-sectional SEM image, indicating that the GaN film with PDMS attached to the surface was lifted off. Although the film was detached from the substrate, it was neatly separated as it maintained its shape prior to lift-off. Figure 5c shows a top-view SEM image of the TPSS following the CLO process. Only GaN was selectively etched because there were only a few residues on some of the lenses. Because no material other than the substrate itself was present, the substrate can be reused, which is an advantage. Figure 5d shows a tilted SEM image of the bottom of GaN, where the air tunnel was located. The interface where GaN nucleation occurred had a rough surface, owing to the damage caused by chemical etching. Because GaN was grown using the ELOG technique, the bottom surface of GaN was cone-shaped. Furthermore, the cone-shaped GaN pattern corresponds to the pattern spacing and position of the TPSS. Thus, the KOH etchant effectively penetrated the air tunnel and etched the interface between the TPSS and GaN. We expect that the structure of the GaN template containing the air tunnel will enable rapid CLO via effective etchant injection.

## 4. Conclusions

An air-tunnel structure was successfully formed by exposing only the c-plane of the TPSS lens, owing to the carbonization of the photoresist before the selective area growth of the GaN layer. The air tunnel maintained its structure under the GaN layer, even though the photoresist gradually disappeared during ELOG at high temperatures. The epitaxial growth of GaN (0002) was confirmed by XRD. The PL spectra of the GaN templates with and without an air tunnel exhibited near-band edge luminescence with an intense peak at 364 nm. In addition, broadband yellow luminescence that spanned 500–600 nm was observed in the case of the GaN template with an air tunnel, indicating internal GaN structural defects. Both samples, which appeared to have compressive stress compared with free-standing GaN, showed redshifted Raman spectroscopy results. Carbon, a byproduct generated during carbonization, is considered to have caused dislocation or internal stress in the GaN film with the air tunnel compared with the GaN film without the air tunnel. We successfully separated the substrate from GaN via CLO to overcome the limitations of laser lift-off. CLO minimizes the defects in the grown GaN layer but has the disadvantage of requiring a long time. Therefore, we created a GaN template with an air-tunnel structure using the TPSS. The KOH solution penetrated the air tunnel and etched the interface between GaN and the TPSS in 5 h, evenly separating the template from substrate.

## Data Availability

Not applicable.

## References

[1] DenBaars S.P., Feezell D., Kelchner K., Pimputkar S., Pan C.-C., Yen C.-C., Tanaka S., Zhao Y., Pfaff N., Farrell R. (2013). Development of Gallium-Nitride-Based Light-Emitting Diodes (LEDs) and Laser Diodes for Energy-Efficient Lighting and Displays. Acta Mater..

[2] Griffiths A.D., Herrnsdorf J., McKendry J.J.D., Strain M.J., Dawson M.D. (2020). Gallium Nitride Micro-Light-Emitting Diode Structured Light Sources for Multi-Modal Optical Wireless Communications Systems. Phil. Trans. R. Soc. A.

[3] Nakamura S., Mukai T., Senoh M. (1991). High-Power GaN P-N Junction Blue-Light-Emitting Diodes. Jpn. J. Appl. Phys..

[4] Beaumont B., Gibart P., Vaille M., Haffouz S., Nataf G., Bouillé A. (1998). Lateral Overgrowth of GaN on Patterned GaN/Sapphire Substrate via Selective Metal Organic Vapour Phase Epitaxy: A Route to Produce Self Supported GaN Substrates. J. Cryst. Growth.

[5] Yamada M., Mitani T., Narukawa Y., Shioji S., Niki I., Sonobe S., Deguchi K., Sano M., Mukai T. (2002). InGaN-Based Near-Ultraviolet and Blue-Light-Emitting Diodes with High External Quantum Efficiency Using a Patterned Sapphire Substrate and a Mesh Electrode. Jpn. J. Appl. Phys..

[6] Gao H., Yan F., Zhang Y., Li J., Zeng Y., Wang G. (2008). Enhancement of the Light Output Power of InGaN/GaN Light-Emitting Diodes Grown on Pyramidal Patterned Sapphire Substrates in the Micro- and Nanoscale. J. Appl. Phys..

[7] Lee Y.J., Hsu T.C., Kuo H.C., Wang S.C., Yang Y.L., Yen S.N., Chu Y.T., Shen Y.J., Hsieh M.H., Jou M.J. (2005). Improvement in Light-Output Efficiency of near-Ultraviolet InGaN–GaN LEDs Fabricated on Stripe Patterned Sapphire Substrates. Mater. Sci. Eng. B.

[8] Lee Y.-J., Chiu C.-H., Ke C.C., Lin P.C., Lu T.-C., Kuo H.-C., Wang S.-C. (2009). Study of the Excitation Power Dependent Internal Quantum Efficiency in InGaN/GaN LEDs Grown on Patterned Sapphire Substrate. IEEE J. Sel. Top. Quantum Electron..

[9] Dai Q., Schubert M.F., Kim M.H., Kim J.K., Schubert E.F., Koleske D.D., Crawford M.H., Lee S.R., Fischer A.J., Thaler G. (2009). Internal Quantum Efficiency and Nonradiative Recombination Coefficient of GaInN/GaN Multiple Quantum Wells with Different Dislocation Densities. Appl. Phys. Lett..

[10] Shin D.-S., Han D.-P., Oh J.-Y., Shim J.-I. (2012). Study of Droop Phenomena in InGaN-Based Blue and Green Light-Emitting Diodes by Temperature-Dependent Electroluminescence. Appl. Phys. Lett..

[11] Kato Y., Kitamura S., Hiramatsu K., Sawaki N. (1994). Selective Growth of Wurtzite GaN and Al_x_Ga_1−x_N on GaN/Sapphire Substrates by Metalorganic Vapor Phase Epitaxy. J. Cryst. Growth.

[12] Tanikawa T., Rudolph D., Hikosaka T., Honda Y., Yamaguchi M., Sawaki N. (2008). Growth of Non-Polar (112¯0)GaN on a Patterned (110)Si Substrate by Selective MOVPE. J. Cryst. Growth.

[13] Zheleva T.S., Nam O.-H., Bremser M.D., Davis R.F. (1997). Dislocation Density Reduction via Lateral Epitaxy in Selectively Grown GaN Structures. Appl. Phys. Lett..

[14] Melton W.A., Pankove J.I. (1997). GaN Growth on Sapphire. J. Cryst. Growth.

[15] Jang S., Lee D., Kwon J.-H., Kim S.-I., Yim S.Y., Lee J., Park J.H., Byun D. (2012). Study of A-Plane GaN Epitaxial Lateral Overgrowth Using Carbonized Photoresist Mask on r-Plane Sapphire. Jpn. J. Appl. Phys..

[16] Song Y.-K., Diagne M., Zhou H., Nurmikko A.V., Carter-Coman C., Kern R.S., Kish F.A., Krames M.R. (1999). A Vertical Injection Blue Light Emitting Diode in Substrate Separated InGaN Heterostructures. Appl. Phys. Lett..

[17] Wong W.S., Sands T., Cheung N.W. (1998). Damage-Free Separation of GaN Thin Films from Sapphire Substrates. Appl. Phys. Lett..

[18] Cao X.A., Arthur S.D. (2004). High-Power and Reliable Operation of Vertical Light-Emitting Diodes on Bulk GaN. Appl. Phys. Lett..

[19] Tan B.S., Yuan S., Kang X.J. (2004). Performance Enhancement of InGaN Light-Emitting Diodes by Laser Lift-off and Transfer from Sapphire to Copper Substrate. Appl. Phys. Lett..

[20] Chu C.-F., Lai F.-I., Chu J.-T., Yu C.-C., Lin C.-F., Kuo H.-C., Wang S.C. (2004). Study of GaN Light-Emitting Diodes Fabricated by Laser Lift-off Technique. J. Appl. Phys..

[21] Delmdahl R., Pätzel R., Brune J. (2013). Large-Area Laser-Lift-Off Processing in Microelectronics. Phys. Procedia.

[22] Chen M., Zhang J.-Y., Lv X.-Q., Ying L.-Y., Zhang B.-P. (2013). Effect of Laser Pulse Width on the Laser Lift-off Process of GaN Films. Chin. Phys. Lett..

[23] Chuang S.-H., Pan C.-T., Shen K.-C., Ou S.-L., Wuu D.-S., Horng R.-H. (2013). Thin Film GaN LEDs Using a Patterned Oxide Sacrificial Layer by Chemical Lift-Off Process. IEEE Photon. Technol. Lett..

[24] Hsueh H.-H., Ou S.-L., Wuu D.-S., Horng R.-H. (2015). InGaN LED Fabricated on Eco-GaN Template with a Ga2O3 Sacrificial Layer for Chemical Lift-off Application. Vacuum.

[25] Cheng C.-W., Shiu K.-T., Li N., Han S.-J., Shi L., Sadana D.K. (2013). Epitaxial Lift-off Process for Gallium Arsenide Substrate Reuse and Flexible Electronics. Nat. Commun..

[26] Lin C.-F., Dai J.-J., Lin M.-S., Chen K.-T., Huang W.-C., Lin C.-M., Jiang R.-H., Huang Y.-C. (2010). An AlN Sacrificial Buffer Layer Inserted into the GaN/Patterned Sapphire Substrate for a Chemical Lift-Off Process. Appl. Phys. Express.

[27] Jeong W.S., Ahn M.J., Ko H.-A., Shim K., Kang S., Kim H., Kim D., Jhin J., Byun D. (2023). Fabrication Method of GaN Template for High-Speed Chemical Lift-Off. AIP Adv..

[28] Wang M.-T., Liao K.-Y., Li Y.-L. (2011). Growth Mechanism and Strain Variation of GaN Material Grown on Patterned Sapphire Substrates with Various Pattern Designs. IEEE Photon. Technol. Lett..

[29] Wang H.-Y., Lin Z.-T., Han J.-L., Zhong L.-Y., Li G.-Q. (2015). Design of Patterned Sapphire Substrates for GaN-Based Light-Emitting Diodes. Chin. Phys. B.

[30] Horng R.-H., Hsueh H.-H., Ou S.-L., Tsai C.-T., Tsai T.-Y., Wuu D.-S. (2017). Chemical Lift-off Process for Nitride LEDs from an Eco-GaN Template Using an AlN/Strip-Patterned-SiO_2_ Sacrificial Layer: Chemical Lift-off Process for Nitride LEDs. Phys. Status Solidi A.

[31] He C., Zhao W., Zhang K., He L., Wu H., Liu N., Zhang S., Liu X., Chen Z. (2017). High-Quality GaN Epilayers Achieved by Facet-Controlled Epitaxial Lateral Overgrowth on Sputtered AlN/PSS Templates. ACS Appl. Mater. Interfaces.

[32] Forsberg M., Serban A., Poenaru I., Hsiao C.-L., Junaid M., Birch J., Pozina G. (2015). Stacking Fault Related Luminescence in GaN Nanorods. Nanotechnology.

[33] Junaid M., Chen Y.-T., Palisaitis J., Garbrecht M., Hsiao C.-L., Persson P.O.Å., Hultman L., Birch J. (2015). Liquid-Target Reactive Magnetron Sputter Epitaxy of High Quality GaN(0001⁻) Nanorods on Si(111). Mater. Sci. Semicond. Process..

[34] Ke W.-C., Liang Z.-Y., Tesfay S.T., Chiang C.-Y., Yang C.-Y., Chang K.-J., Lin J.-C. (2019). Epitaxial Growth and Characterization of GaN Thin Films on Graphene/Sapphire Substrate by Embedding a Hybrid-AlN Buffer Layer. Appl. Surf. Sci..

[35] Tang H., Webb J.B., Bardwell J.A., Raymond S., Salzman J., Uzan-Saguy C. (2001). Properties of Carbon-Doped GaN. Appl. Phys. Lett..

[36] Zhou D., Ni Y., He Z., Yang F., Yao Y., Shen Z., Zhong J., Zhou G., Zheng Y., He L. (2016). Investigation of Breakdown Properties in the Carbon Doped GaN by Photoluminescence Analysis. Phys. Status Solidi C.

[37] Senthil Kumar M., Kumar J. (2003). XRD, XPS, SEM, PL and Raman Scattering Analysis of Synthesised GaN Powder. Mater. Chem. Phys..

[38] Minsky M.S., White M., Hu E.L. (1996). Room-temperature Photoenhanced Wet Etching of GaN. Appl. Phys. Lett..

[39] Stocker D.A., Schubert E.F., Redwing J.M. (1998). Crystallographic Wet Chemical Etching of GaN. Appl. Phys. Lett..

